# Comparative evaluation of single or combined anticoccidials on performance, antioxidant status, immune response, and intestinal architecture of broiler chickens challenged with mixed *Eimeria* species

**DOI:** 10.1016/j.psj.2021.101162

**Published:** 2021-03-26

**Authors:** AbdelRahman Y. Abdelhady, Salah A. El-Safty, Mosaad Hashim, Marwa A. Ibrahim, Faten F. Mohammed, Ahmed M. Elbaz, Abdel-Moneim Eid Abdel-Moneim

**Affiliations:** ⁎ Department of Poultry Production, Faculty of Agriculture, Ain Shams University, Hadayek Shoubra 11241, Cairo, Egypt; † Applied Feed Research House (AFRH), Orabi Community, Qalyobia, Egypt; ‡ Biochemistry Department and Molecular Biology, Faculty of Veterinary Medicine, Cairo University, Giza, Egypt; § Department of Pathology, Faculty of Veterinary Medicine, Cairo University, Giza, 12211, Egypt; # Desert Research Center, Mataria, Cairo, Egypt; ‖ Biological Applications Department, Nuclear Research Center, Egyptian Atomic Energy Authority, Abu-Zaabal 13759, Egypt

**Keywords:** anticoccidials, performance, antioxidant, immunity, broiler

## Abstract

Poultry production faces several threats and challenges, one of the most important of which is avian coccidiosis which causes annual losses exceeding US$ 3 billion. Discovering new drugs or combinations of existing anticoccidials has become inevitable to overcome the emergence of coccidiosis resistance. This study evaluated a new combination of maduramicin and diclazuril in comparison to the well-known product Maxiban72 which consisted of narasin and nicarbazin, and the single effect of monensin as treatments for avian coccidiosis. A total of 750 1-day-old Indian River broiler chicks were allocated equally into 5 experimental groups with 6 replicates each as follows: 1) negative unchallenged control group **(NC)** fed the basal diet; 2) positive control group **(PC)** received the basal diet and inoculated with *Eimeria*; 3) PC + 100 mg monensin sodium (Atomonsin)/kg diet **(MS)**; 4) PC + 5 mg maduramicin ammonium (Madramycin) + 2.5 mg diclazuril (Atozuril)/kg diet **(MMD)**; and 5) PC + 40 mg narasin + 40 mg nicarbazin (Maxiban^T^72)/kg diet **(NN)**. Anticoccidials improved (*P* < 0.01) growth performance, dressing (%) and carcass yield of inoculated birds compared to untreated-inoculated ones. Erythrogram and leukogram parameters were affected by Eimeria challenge. Total protein, globulin, cholesterol, triglycerides, superoxide dismutase and glutathione peroxidase levels in PC birds' serum were reduced (*P* < 0.05) while their values of liver enzymes, malondialdehyde and catalase were elevated (*P* < 0.01) when compared to NC ones. Serum immunoglobulin A, and jejunal gene expressions of interleukin-6 and interferon gamma were increased (*P* < 0.05) in PC group compared to NC group. Anticoccidial drugs restored values of the aforementioned biomarkers near to those of NC. Jejunal architecture in inoculated birds was improved by the anticoccidial treatments in MS, MMD, and NN. Fecal oocyst counts were significantly reduced in MMD, NN, and MS groups compared to PC group. Conclusively, although all examined anticoccidial drugs were effective in treating Eimeriosis, the anticoccidial combinations in MMD and NN groups were more effective than the single administration of MS in treating avian coccidiosis.

## INTRODUCTION

Poultry meat occupies a distinguished position as an important meat source around the world, with a total production of approximately 120 billion tons annually, which represents more than one-third of the humans' protein food and is expected to double by 2050 (Alexandratos and Bruinsma, 2012). However, avian coccidiosis, caused by the parasite *Eimeria* protozoan, is considered one of the main endemic threats to birds' production, causing annual economic losses exceed US$ 3 billion (Noack et al., 2019). Therefore, avian coccidiosis must be controlled if poultry meat is to be relied upon to fulfill the growing global demand for protein. Economic losses of infected birds are associated with poor performance and increased mortality caused by numerous disturbances in the physiological and metabolic homeostasis of these birds (Abd El‐Hack et al., 2020; Abd El-Hack et al., 2021). Coccidiosis alters digestion and absorption of nutrients (Major Jr and Ruff, 1978; Adams et al., 1996; Su et al., 2015), expression of genes encoding transport proteins and digestive enzymes in the small intestine (Su et al., 2015; Miska and Fetterer, 2018), as well as intestinal morphology (Morris et al., 2004; Gottardo et al., 2016; Abdelhady et al., 2020). The antioxidant (Bun et al., 2011) and immune systems (Lillehoj and Trout, 1996) are also activated during the infection because of the increase in the reactive oxygen **(ROS)** and nitrogen species formation (Allen, 1997), the reduction in non-enzymatic antioxidants concentrations (Allen and Fetterer, 2002) and the alterations in activities of antioxidant enzymes (Georgieva et al., 2006; Bun et al., 2011). In addition, coccidial infections compromise animal welfare and food safety as well (Kadykalo et al., 2018). Thereby, to achieve sustainable poultry production, control of the infection using anticoccidial drugs is indispensable.

Prophylaxis method to constrain avian coccidiosis using anticoccidial chemicals, coccidiostats, coccidiocides, ionophores and live attenuated or non-attenuated vaccines is the current successful and cost-effective approach in modern poultry production since once clinical signs appear, treatments are often too late to prohibit the infection's pathological consequences (Chapman, 2009; Muthamilselvan et al., 2016). In general, anticoccidial drugs belong to one of 2 classes: ionophores (polyether antibiotics) and synthetic chemicals (Chapman, 1997). Fermentation of *Actinomadura spp.* or *Streptomyces spp.* are used to produce ionophores which consist of 3 types. These types are monovalent glycosidic ionophores (semduramicin, maduramicin), monovalent ionophores (salinomycin, monensin, narasin), and divalent ionophore (lasalocid) (Noack et al., 2019). These drugs able to disrupt the ion gradients across the parasite cell membrane while synthetic chemicals eliminate coccidiosis with one or more of the following specific mode of action: 1) suppression of the folic acid pathway (sulfonamides), 2) inhibition of thiamine uptake competitively (amprolium), 3) inhibition of mitochondrial respiration of the parasite (decoquinate, clopidol), or 4) unknown mode of action (e.g., nicarbazin, diclazuril, robenidine, halofuginone) (Noack et al., 2019). Due to the chemoprophylactic control of coccidiosis using these synthetic anticoccidials and ionophores, the resistance for these drugs, which have been permitted for use in poultry, has been noticed (Chapman, 1997). Hence, discovering new drugs has became inevitable, but efforts have been undertaken in this area are very limited and no novel chemical drugs have been introduced for decades (Chapman et al., 2013). Fortunately, the emergence of coccidiosis resistance can be slacken using different ionophores and/or chemicals in rotation programs (Chapman et al., 2013). Additionally, it has been reported that avian coccidiosis may not be treated or controlled using only one compound but it requires the combination of synthetic chemicals and ionophores that can interfere with *Eimeria* life cycle or destroy its oocysts (Quiroz-Castañeda, 2018). Therefore, the present study evaluated the single effect of monensin (Atomonsin) and the impact of a new combination consisting of maduramicin (Madramycin) and diclazuril (Atozuril) in comparison to the well known product Maxiban72 which consisted of narasin and nicarbazin as treatments for broiler chickens infected with mixed *Eimeria* species.

## MATERIALS AND METHODS

The present experiment was conducted at Poultry Production Farm, Applied Feed Research House (AFRH), Orabi Community, Qalyobia Governorate, Egypt. Animal protocols were approved by Animal Care and Welfare Committee at Faculty of Agriculture, Ain Shams University, Egypt.

### Birds, Deits, and Management Practices

A total of 750 one-day-old Indian River broiler chicks were obtained from a local commercial hatchery, weighed upon arrival and randomly allocated into 5 experimental groups with 6 replicates (pens)/group containing 25 chicks each. The pens with surface area of 2 m^2^ were littered with wood shavings. Shed temperature was kept at 34° to 31°C during the first week of age and was gradually decreased by 3°C/wk until reaching 26°C. All birds were kept under uniform management conditions in a well-ventilated shed. Boots and clothing were replaced before entering the unchallenged pens which were monitored first prior to attending to challenged birds. Feed and drinking water were offered ad libitum. Birds were fed with corn-soybean meal-based starter (1–21 D) and grower (22–35 D) diets (Table 1) which were formulated as recommended by Indian River strain catalogue (Aviagen, 2019). The composition and calculated analysis of the diets are shown in Table 1. The groups consisted of 1) negative control group (**NC**) received the basal diet and not inoculated with *Eimeria*; 2) positive control group (**PC**) received the basal diet and inoculated with *Eimeria*; 3) PC supplemented with 100 mg monensin sodium (Atomonsin)/kg diet (**MS**); 4) PC supplemented with 5 mg maduramicin ammonium (Madramycin) + 2.5 mg diclazuril (Atozuril)/kg diet (**MMD**); and 5) PC supplemented with 40 mg narasin + 40 mg nicarbazin (Maxiban72)/kg diet (**NN**). On d 3, each chick in NC group were orally gavaged a 1 mL distilled water while chicks of PC, MS, MMD, and NN groups were orally gavaged equal volume with a 50 × commercial coccidosis vaccine dose (Coccivac-D, Intervet Inc., Omaha). The vaccine provided a mixture of live oocysts of *Eimeria tenella, E. brunetti, E. hagani, E. mivati, E. acervulina, E. maxima, E. necatrix, and E. praecox*.

**Table 1:** Ingredients and calculated chemical composition of the basal diet.

Ingredients	Starter (1–21 d)	Grower (22–35 d)
Yellow corn, %	54.03	58.98
Soybean meal (44%), %	34.50	29.50
Corn germ (62%), %	5.50	5.50
Soya oil, %	1.80	2.30
Limestone, %	1.08	0.95
Di-Calcium Phosphate, %	2.00	1.75
Premix^1^, %	0.30	0.30
NaCl, %	0.30	0.30
L-lysine, %	0.29	0.24
DL-Methionine, %	0.20	0.18
Calculated composition^2^**(%)**		
Metabolizable energy (ME, kcal kg^−1^)	3001	3180
Crude protein	23.12	20.99
Calcium	0.99	0.89
Potassium	0.54	0.52
Available Phosphorus	0.51	0.46
Digestible methio + Cys	0.93	0.89
Digestible methionine	0.59	0.52
Digestible lysine	1.43	1.24
Digestible arginine	1.25	1.07
Digestible tryptophan	0.19	0.17

1 Provides each kg of diet: Vit. A: 12000 IU, Vit. D_3_: 5000 IU, Vit. E: 130.0 mg, Vit. K_3_:3.61 mg, Vit. B_1_:3.0 mg, Vit. B_2_:8.0 mg, Vit. B_6_:4.95 mg, Vit. B_12_: 0.17 mg, Niacin:60.0 mg, Folic acid:2.08 mg, D-Biotin:200.0 mg, calcium D-Pantothenate: 18.33 mg, Copper:80.0 mg, Iodine:2.0 mg, Selenium:150.0 mg, Iron:80.0 mg, Manganese:100.0 mg, Zinc:80.0 mg, Cobalt: 500.0 mg.

2 Calculated according to Aviagen (2019).

### Performance Parameter and Histology

At 21 and 35 ds of age, body weight **(BW)** was recorded at early morning and feed intake was measured by subtracting the feed residue from the feed provided. Weight gain **(WG)** was measured as a difference of weight between 2 weighing intervals. Feed conversion ratio was calculated as g feed/g gain per period and adjusted for mortality. At 35 d of age, prior slaughtering, 6 birds per group (one bird per pen) were weighed and necropsied. Hot carcass, liver, gizzard, heart, spleen, thymus and bursa of Fabricius were weighed and estimated as a percentage of live BW. Dressing percentage and carcass yield were estimated as described by Abd El-Moneim et al. (2020) and Abdel-Moneim et al. (2020a). Abdominal fats were collected from the abdominal and pelvic cavities and weighed. The average of 2 thighs and breast (with the sternum) were also weighed and proportional to live BW (Abo Ghanima et al., 2020a). Two samples from jejunum from each bird were collected. The first sample was immediately kept in liquid nitrogen and stored at −80°C until real-time PCR analysis. The other sample was fixed in a 10% formalin-saline solution, dehydrated, cleared, embedded in paraffin wax and blocked. Sections of 4 to 5 µm thickness were cut using rotary microtome and stained with Haematoxylline and eosin for general morphometry examinations under light microscope (Bancroft and Layton, 2019).

### Blood Hematological and Biochemical Indices

At the end of the experiment, 2 blood samples were collected from the wing vein in separate labeled tubes from 6 birds per group (one bird/pen). One of these tubes contained EDTA for erythrogram (Erythrocytes count, hemoglobin **[Hb]** concentration, and hematocrit **[PCV**]) and leukogram (leukocytes, lymphocytes, heterophils, monocytes, basophils, and eosinophils counts) assays which were measured as described by Abo Ghanima et al. (2020b). The second blood sample was left to clot and centrifuged at 3500 × *g* for 15 min to separate sera samples which were stored at −20°C until the biochemical analysis. Total protein **(TP)**, albumin, globulin, alkaline phosphatase **(ALP)**, alanine aminotransferase **(ALT)**, aspartate aminotransferase **(AST)**, triglycerides **(TG)**, total cholesterol **(TC)**, high-density lipoprotein **(HDL)**, low-density lipoprotein **(LDL)**, and very low-density lipoprotein **(VLDL)** were spectrophotometrically determined (Spectronic 1201; Milton Roy, Ivyland, PA) using commercial kits (Cell Biolabs Inc., San Diego, CA) according to the instructions of the manufacturer.

Oxidative stress biomarkers, including malondialdehyde **(MDA)** content, catalase, superoxide dismutase **(SOD)**, and glutathione peroxidase **(GPx)** activities were determined in sera samples using the ELISA kit from Quanti Chrom, BioAssay Systems, USA and Cayman Chemical Company, USA. In appropriately diluted samples, serum immunoglobulin **(Ig)** A concentration was assayed using chicken-specific IgA ELISA quantitation kits and microtiter plates (Bethyl Laboratories Inc., Montgomery, TX) by a sandwich ELISA according to the manufacturer's protocol (Abdel-Moneim et al., 2020b). The absorbance was measured at 450 nm.

### Fecal Coccidial Oocyst Shedding

On d 34, trays were placed in each pen for excreta collection. Excreta samples were collected at 35 d of age from each pen and immediately mixed, pooled into one sample, placed in numbered plastic bags, refrigerated at 4°C and transported to the laboratory for further analysis. *Eimeria* oocysts were quantified as described by Arendt et al. (2016) using McMaster technique.

### Quantitative Real-Time PCR

Using RNeasy mini kit (Qiagen), total RNA was isolated from jejunal tissue samples according to the manufacturer's instructions. By reverse transcription of 10μg RNA samples, first-strand cDNA was generated (Abdou K et al., 2019). The primer set used for Glutathione peroxidase *(GPx)* (NM_001277853.2) are Forward primer: GATGACCAACCCGCAGTACA; Reverse primer: GCTTTGAAAACATCGGGCG, Superoxide dismutase (*SOD*) (NM_204211.1) Forward: TACAGCTCAGGTGTCGCTTC; Reverse: GCGAAGGAACCAAAGTCACG, Interferon-gamma *(IFN-γ)* (NM_205149.1) Forward: TGTAGCTGACGGTGGACCT; Reverse: ATGTGTTTGATGTGCGGCTT and those of the interleukin-6 *(IL-6)* (NM_204628.1) were Forward primer: CTGCAGGACGAGATGTGCAA; Reverse primer: AGGTCTG AAAGGCGAA CAGG. Real-time PCR was run for the four genes for 40 cycles of denaturation at 95°C for 30 s, annealing at 60°C for 30 s, and extension at 72°C for 30 s using a Real-Time PCR System (Applied Biosystems, USA). The β-actin gene, which served as the internal control, was amplified in the same reaction (Hassanen et al., 2019). Each assay was repeated 3 times, and the gene/β-actin ratio was calculated. Mxpro software was used to calculate the normalized expression ratio (Morgan et al., 2019).

### Statistical Analysis

Data were analyzed using One-way analysis of variance, General Linear Model's procedure (SPSS 19, 2018). Identification of significance (*P* < 0.05) among multiple means was performed using Tukey's multiple comparison test.

## RESULTS

### Growth Performance

BW and WG of PC group at 21 and 35 days of age were reduced (*P* < 0.001) compared to NC group (Table 2). Administration of anticoccidial combinations in MMD and NN groups enhanced (*P* < 0.001) the values of the aforementioned parameters better than the single inclusion of MS. Untreated challenged birds consumed less feed (*P* < 0.05) than the rest of experimental groups during all the experimental periods. Feed conversion ratio during the grower and overall periods was not significantly altered among experimental groups.

**Table 2:** Effect of dietary anticoccidial substances on growth performance of broiler chickens infected with mixed *Eimeria* species.

Items^1^	Body weight, g			Weight gain, g.bird.day^−1^			Feed intake, g.bird.day^−1^			Feed conversion ratio, g feed.g gain^−1^		
	Initial	21 d	35 d	1–21 d	22–35 d	1–35 d	1–21 d	22–35 d	1–35 d	1–21 d	22–35 d	1–35 d
NC	40.40	1033.3^a^	2243.7^a^	47.28^a^	86.45^a^	62.95^a^	54.63^a^	148.1^a^	92.01^a^	1.16^c^	1.73	1.46
PC	39.72	868.7^b^	1907.1^c^	39.48^b^	74.17^b^	53.35^c^	49.64^b^	127.6^b^	80.82^b^	1.26^b^	1.72	1.51
MS	40.04	872.0^b^	2032.3^b^	39.62^b^	82.88^a^	56.92^b^	54.06^a^	143.5^a^	89.82^a^	1.37^a^	1.73	1.58
MMD	39.84	913.9^b^	2177.6^a^	41.63^b^	90.27^a^	61.08^a^	56.50^a^	146.1^a^	92.33^a^	1.36^a^	1.62	1.51
NN	40.20	910.3^b^	2145.7^a^	41.44^b^	88.24^a^	60.16^a^	54.44^a^	141.0^a^	89.06^a^	1.31^ab^	1.60	1.48
SEM^2^	0.192	12.97	28.36	0.617	1.550	0.810	0.606	1.872	0.997	0.018	0.032	0.017
P-value	0.816	<0.001	<0.001	<0.001	0.001	<0.001	<0.001	0.024	0.004	0.001	0.393	0.259

1 NC = unchallenged group; PC = challenged group; MS= PC + 100 mg Monensin sodium (Atomonsin)/kg diet; MMD= PC + 5 mg Maduramicin ammonium (Madramycin) + 2.5 mg Diclazuril (Atozuril)/kg diet; NN= PC + 40 mg Narasin + 40 mg Nicarbazin (Maxiban**^T^**72)/kg diet.

2 SEM, Standard error of means. Mean in the same row within each classification bearing different letters are significantly different.

Liver, gizzard, heart, thigh, breast, spleen, thymus and abdominal fat relative weights were not statistically affected by *Eimeria* challenge or anticoccidial treatments (Table 3). Dressing (%), carcass yield and bursa of Fabricius relative weight were decreased (*P* < 0.01) in PC group, while the treatment with anticoccidial combinations in MMD and NN groups restored their values to those of NC group.

**Table 3:** Effect of dietary anticoccidial substances on carcass traits (%) of 35-day-old broiler chickens infected with mixed *Eimeria* species.

Items^1^	NC	PC	MS	MMD	NN	SEM	*P*-value
Dressing	73.97^a^	70.99^c^	73.00^b^	74.07^a^	74.11^a^	0.313	<0.001
Liver	3.13	3.36	3.35	3.24	3.44	0.101	0.926
Gizzard	1.51	1.48	1.42	1.44	1.48	0.062	0.996
Heart	0.686	0.695	0.633	0.702	0.648	0.027	0.932
Breast	20.92	18.99	20.01	20.96	20.23	0.343	0.377
Thigh	15.30	13.63	13.77	15.12	15.63	0.331	0.174
Abdominal fat	1.11	1.388	1.789	1.223	1.332	0.128	0.582
Spleen	0.165	0.126	0.178	0.236	0.208	0.014	0.082
Thymus	0.175	0.149	0.299	0.199	0.165	0.022	0.190
Bursa of Fabricius	0.122^a^	0.067^b^	0.069^b^	0.129^a^	0.103^a^	0.008	0.001
Carcass yield	78.63^ab^	75.83^c^	77.77^b^	78.68^ab^	78.91^a^	0.325	<0.001

Abbreviations: SEM, Standard error of means.

Mean in the same row within each classification bearing different letters are significantly different

1 NC = unchallenged group; PC = challenged group; MS= PC + 100 mg Monensin sodium (Atomonsin)/kg diet; MMD= PC + 5 mg Maduramicin ammonium (Madramycin) + 2.5 mg Diclazuril (Atozuril)/kg diet; NN= PC + 40 mg Narasin + 40 mg Nicarbazin (Maxiban72)/kg diet.

### Hematological Parameters

Erythrogram parameters were negatively influenced by *Eimeria* challenge or anticoccidial treatments (Table 4). Erythrocytes and Hb values were decreased (*P* < 0.05) in all challenged birds compared to NC chicks. PCV was decreased significantly (*P* < 0.05) in PC and MS groups and numerically in MMD and NN compared to NC. Mean corpuscular volume, mean corpuscular hemoglobin, and mean corpuscular hemoglobin concentration were not altered among the experimental groups.

**Table 4:** Effect of dietary anticoccidial substances on the erythrogram parameters of 35-day-old broiler chickens infected with mixed *Eimeria* species.

Items^1^	Hb, g.dl^−1^	Erythrocytes, 10^6^.μL	PCV, %	MCV, fl	MCH, pg	MCHC, %
NC	9.58^a^	4.34^a^	37.05^a^	85.64	22.29	25.93
PC	7.11^c^	2.63^c^	29.90^c^	114.8	27.46	23.86
MS	7.93^bc^	3.13^bc^	32.64^bc^	104.6	25.53	24.30
MMD	8.87^ab^	3.51^b^	34.05^ab^	99.10	26.07	26.06
NN	8.09^bc^	3.36^bc^	33.81^ab^	101.5	24.37	23.88
SEM	0.275	0.176	0.739	3.757	1.188	0.526
*P*-value	0.016	0.008	0.011	0.167	0.769	0.532

Abbreviations: Hb, hemoglobin; MCH, mean corpuscular hemoglobin; MCHC, mean corpuscular hemoglobin concentration; MCV, mean corpuscular volume;PCV, hematocrit; SEM, Standard error of means.

Mean in the same row within each classification bearing different letters are significantly different.

1 NC = unchallenged group; PC = challenged group; MS= PC + 100 mg Monensin sodium (Atomonsin)/kg diet; MMD= PC + 5 mg Maduramicin ammonium (Madramycin) + 2.5 mg Diclazuril (Atozuril)/kg diet; NN= PC + 40 mg Narasin + 40 mg Nicarbazin (Maxiban72)/kg diet.

Leukogram parameters were greatly altered among the experimental groups (Table 5). Total leucocytes count, and lymphocytes count and percentage were raised (P < 0.01) in all challenged birds when compared to NC ones. Heterophils (%) was decreased (*P* < 0.05) in treated groups while its number was significantly elevated (*P* < 0.05) only in PC group. Eosinophils (%) was decreased (*P* < 0.01) only in PC group while its count, and the counts and proportions of monocytes and basophils were not significantly changed among the challenged and NC groups.

**Table 5:** Effect of dietary anticoccidial substances on the leukogram parameters of 35-day-old broiler chickens infected with mixed *Eimeria* species.

Items^1^		NC	PC	MS	MMD	NN	SEM	*P*-value
Leukocytes, 10^3^.μL		19.41^c^	30.72^a^	27.52^ab^	25.50^b^	26.60^b^	1.096	0.001
Lymphocytes	%	49.34^b^	59.78^a^	56.48^a^	57.28^a^	57.71^a^	1.026	<0.001
	10^3^.μL	9.59^c^	18.38^a^	15.55^b^	14.59^b^	15.31^b^	0.806	<0.001
Heterophils	%	36.57^a^	33.18^b^	31.93^b^	31.25^b^	30.90^b^	0.671	0.017
	10^3^.μL	7.08^b^	10.20^a^	8.78^ab^	7.99^b^	8.25^b^	0.344	0.025
Monocytes	%	7.95^a^	4.35^c^	6.07^b^	6.10^b^	5.99^b^	0.337	0.001
	10^3^.μL	1.54	1.33	1.67	1.56	1.60	0.058	0.482
Basophils	%	0.243	0.187	0.287	0.217	0.193	0.016	0.276
	10^3^.μL	0.048	0.057	0.079	0.055	0.050	0.004	0.148
Eosinophils	%	5.90^a^	2.50^b^	5.24^a^	5.18^a^	5.20^a^	0.347	0.001
	10^3^.μL	1.15^ab^	0.76^b^	1.44^a^	1.33^a^	1.39^a^	0.084	0.034

Abbreviations: SEM, Standard error of means.

Mean in the same row within each classification bearing different letters are significantly different.

1 NC = unchallenged group; PC = challenged group; MS = PC + 100 mg Monensin sodium (Atomonsin)/kg diet; MMD = PC + 5 mg Maduramicin ammonium (Madramycin) + 2.5 mg Diclazuril (Atozuril)/kg diet; NN = PC + 40 mg Narasin + 40 mg Nicarbazin (Maxiban72)/kg diet.

### Blood Biochemical Indicies

Serum biochemical parameters as affected by Eimeria challenge or anticoccidial treatments are showed in Table 6. Concentrations of TP, globulin, TC, TG, HDL, and VLDL in PC birds serum were reduced (P < 0.05) while their values of AST, ALT and ALP were elevated (P < 0.01) when compared to NC ones. The treatment with anticoccidials either single or in combination restored the values of the abovementioned indicies near to the normal values of NC birds. Anticoccidial combination in MMD and NN groups was more effective than the single adminestration of MS in restoring those values. Albumin, uric acid, creatinine, and LDL were not significantly affected.

**Table 6:** Effect of dietary anticoccidial substances on blood biochemical indices of 35-day-old broiler chickens infected with mixed *Eimeria* species.

Items^1^	NC	PC	MS	MMD	NN	SEM	*P*-value
**Protein fractions, g.dl^−1^**							
Total protein	4.91^a^	2.97^c^	3.80^bc^	4.02^ab^	3.65^bc^	0.203	0.014
Albumin	2.34	1.32	1.70	1.76	1.33	0.139	0.100
Globulin	2.57^a^	1.65^c^	2.10^b^	2.26^ab^	2.32^ab^	0.092	0.002
**Hepatic enzyme activity, U.l^−1^**							
ALT	25.24^c^	87.84^a^	50.50^b^	51.02^b^	61.49^b^	5.567	<0.001
AST	137.5^c^	196.9^a^	168.0^b^	154.3^b^	152.9^bc^	5.667	<0.001
ALP	359.0^c^	871.7^a^	541.0^bc^	447.3^bc^	601.6^b^	52.38	0.002
**Renal function biomarkers, mg.dl^−1^**							
Creatinine	0.613	0.803	0.733	0.680	0.763	0.034	0.461
Uric acid	2.97	6.20	4.63	4.01	4.55	0.403	0.129
**Lipid profile, mg.dl^−1^**							
Total cholesterol	247.3^a^	186.0^d^	191.2^cd^	229.3^ab^	215.7^bc^	6.968	0.003
Triglycerides	221.0^a^	113.3^c^	200.7^a^	194.0^ab^	159.4^b^	11.14	0.001
HDL- cholesterol	46.72^a^	34.42^b^	38.72^b^	42.03^ab^	40.75^ab^	1.373	0.034
LDL- cholesterol	156.4	128.9	112.5	148.5	143.1	5.696	0.082
VLDL- cholesterol	44.20^a^	22.67^c^	40.13^a^	38.80^ab^	31.87^b^	2.228	0.001

Abbreviations: AST, aspartate aminotransferase; ALT, alanine aminotransferase; ALP, alkaline phosphatase; HDL, high-density lipoprotein; LDL, low-density lipoprotein; SEM, Standard error of means; VLDL, very low-density lipoprotein.

Mean in the same row within each classification bearing different letters are significantly different.

1 NC = unchallenged group; PC = challenged group; MS = PC + 100 mg Monensin sodium (Atomonsin)/kg diet; MMD = PC + 5 mg Maduramicin ammonium (Madramycin) + 2.5 mg Diclazuril (Atozuril)/kg diet; NN = PC + 40 mg Narasin + 40 mg Nicarbazin (Maxiban72)/kg diet.

### Jejunal Histomrphometry and Fecal Coccidial Oocyst Shedding

**Figure 1:**
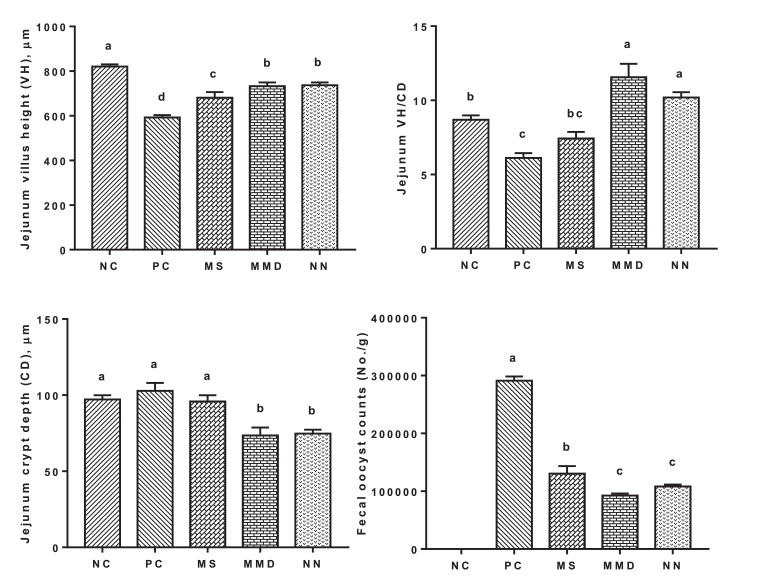
Effect of dietary anticoccidial substances on jejunal histomorphometry and fecal oocyst count of 35-day-old broiler chickens infected with mixed *Eimeria* species. NC, unchallenged group; PC, challenged group; MS, PC + 100 mg Monensin sodium (Atomonsin)/kg diet; MMD, PC + 5 mg Maduramicin ammonium (Madramycin) + 2.5 mg Diclazuril (Atozuril)/kg diet; NN, PC + 40 mg Narasin + 40 mg Nicarbazin (Maxiban72)/kg diet. Data are presented as the mean values with their standard errors. Values with different superscript letters are significantly different (*P* < 0.05).

As illustrated in Figure 1, jejunal villous height **(VH)** was remarkably reduced in PC compared to NC while crypt depth **(CD)** was not affected. Anticoccidial treatments in MS, MMD, and NN improved jejunal architecture in challenged birds; VH was higher in these groups compared to PC, while CD was decreased and VH/CD ratio was increased in MMD and NN groups compared to the rest of groups. Fecal oocyst counts were significantly reduced in MMD, NN and MS groups compared to PC group. Lowest oocyst number was recorded in MMD group followed by NN group.

### Antioxidant Status

**Figure 2:**
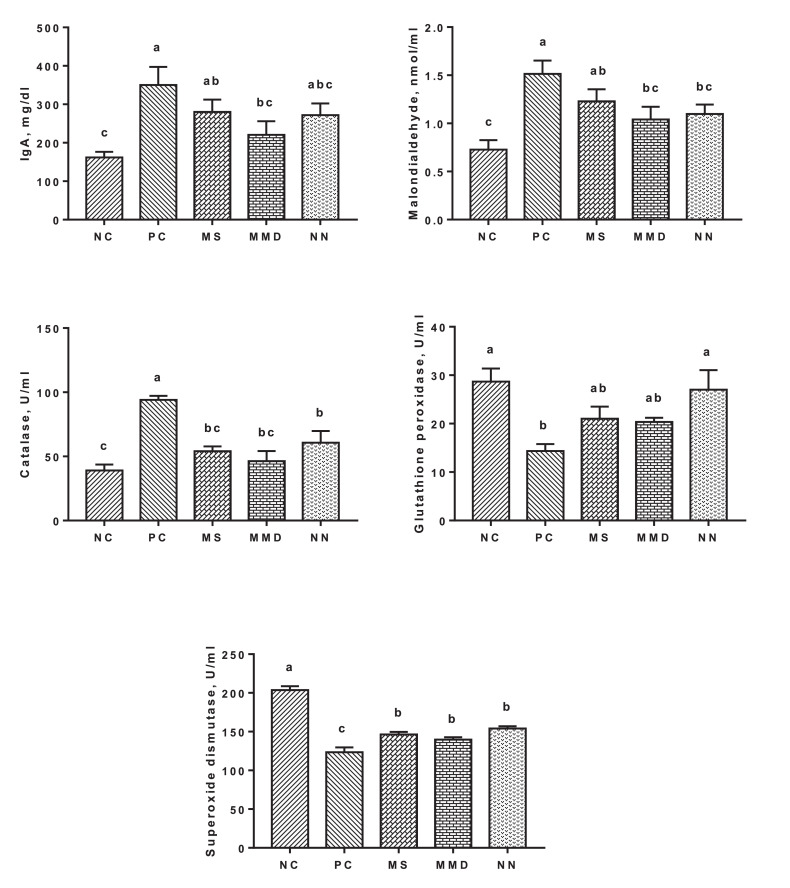
Effect of dietary anticoccidial substances on serum concentrations of immunoglobulin A and oxidative stress biomarkers of 35-day-old broiler chickens infected with mixed *Eimeria* species. NC, unchallenged group; PC, challenged group; MS, PC + 100 mg Monensin sodium (Atomonsin)/kg diet; MMD, PC + 5 mg Maduramicin ammonium (Madramycin) + 2.5 mg Diclazuril (Atozuril)/kg diet; NN, PC + 40 mg Narasin + 40 mg Nicarbazin (Maxiban72)/kg diet. Data are presented as the mean values with their standard errors. Values with different superscript letters are significantly different (*P* < 0.05).

**Figure 3:**
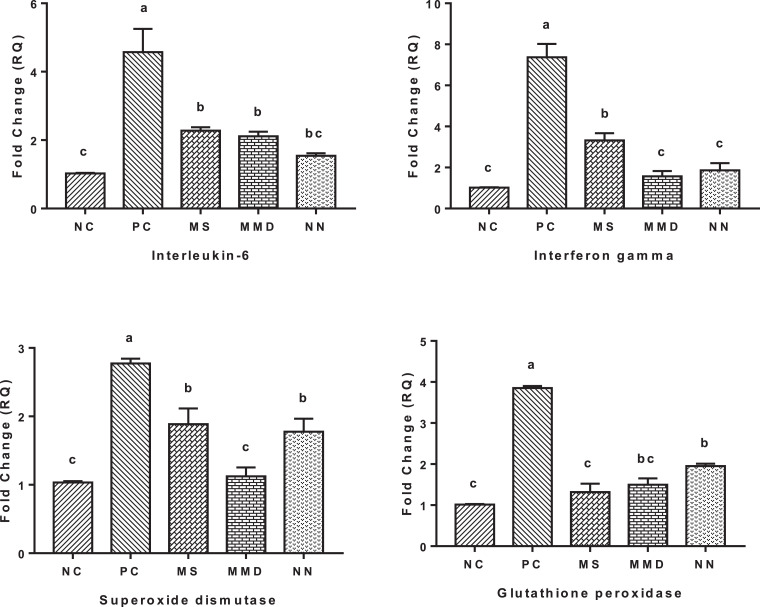
Effect of dietary anticoccidial substances on m-RNA relative quantitation level (RQ) of interleukin-6, interferon gamma, superoxide dismutase and glutathione peroxidase in jejunal tissues of 35-day-old broiler chickens infected with mixed *Eimeria* species. NC, unchallenged group; PC, challenged group; MS, PC + 100 mg Monensin sodium (Atomonsin)/kg diet; MMD, PC + 5 mg Maduramicin ammonium (Madramycin) + 2.5 mg Diclazuril (Atozuril)/kg diet; NN, PC + 40 mg Narasin + 40 mg Nicarbazin (Maxiban72)/kg diet. Data are presented as the mean values with their standard errors. Values with different superscript letters are significantly different (*P* < 0.05).

As depicted in Figures 2 and 3, among oxidative stress biomarkers, serum contents of MDA and catalase were elevated (*P* < 0.01) in PC birds compared to unchallenged birds. All anticoccidials were able to reduce catalase value close to those of NC while MDA was only reduced in MMD and NN groups. SOD and GPx values were decreased (*P* < 0.05) in the serum of PC birds. Treatments with anticoccidial substances elevated SOD value significantly in the serum of challenged birds while only NN group was able to restore GPx level close to that of NC. Gene expression of GPx in jejunal tissues was downregulated in MS, MMD, and NN groups compared to those of PC. The same trend was noticed in the m-RNA expression of SOD and MMD group was more effective than NS and NN groups.

### Immune response

Ig A level was elevated (*P* < 0.05) in the serum of PC group compared to NC group (Figure 2). Dietary supplementation of MMD group significantly reduced IgA value while its levels were numerically decreased in MS and NN groups when compared to PC group. Single or combined anticoccidials used in this study were able to downregulate the gene expression of IL-6 and IFN-γ in challenged birds near to NC birds (Figure 3).

## DISCUSSION

Avian coccidiosis is a highly infectious disease which can be transmitted easily by insects, equipment, water, and contaminated diets. *Eimeria* species are responsible for serious economic losses and can be controlled by anticoccidials and live attenuated or non-attenuated vaccines. Anticoccidial drugs belong to one of 2 classes: ionophores (polyether antibiotics) and synthetic chemicals. Ionophores (such as monensin, narasin and maduramicin) have the ability to disrupt the ion gradients across the parasite cell membrane. Nevertheless, the biochemical response of nicarbazin and diclazuril that produce their effects in broilers, despite their use as synthetic chemicals for many years, are poorly understood (Da Costa et al., 2017; El-Shazly et al., 2020). The combination effects between some of these substances are still also unknown. The potential roles of these synthetic chemicals in reducing *Eimeria* population could be through downregulating microneme genes (Zhou et al., 2010) and the expression of serine/threonine protein phosphatase (type 5), which is a crucial regulatory enzyme in cellular replication of *Eimeria* (Zhou et al., 2013). These drugs might also prevent the synthesis of amylopectin that is aminopolysaccharide present in great quantities in the cell walls of *Eimeria* inside the schizonts during the asexual reproduction, thus hindering merozoites formation and eventually inhibiting merozoites exogenesis (Noack et al., 2019). The foregoing explains the reduction in fecal oocyst shedding in challenged birds treated with anticoccidial drugs in NS, MMD and NN groups compared with PC birds (Figure 1).

Deteriorated BW, WG, FI, dressed (%) and carcass yield (%) of PC birds in the present study (Tables 2 and 3) can be explained by the reduction in digestion (Adams et al., 1996; Su et al., 2015), expression of genes encoding transport proteins and digestive enzymes (Su et al., 2015; Miska and Fetterer, 2018), as well as intestinal morphology (Morris et al., 2004; Gottardo et al., 2016) caused by eimeriosis; resulting in the reduction of absorptive surface area, and nutrients malabsorption. The role of anticoccidials in NS, MMD, and NN groups to improve growth performance parameters could be attributed to their ability to reestablish intestinal microbial balance which then stimulates the secretion of endogenous digestive enzymes, enhance gut passage rate and improve intestinal morphology and nutrients utilization (Bozkurt et al., 2016). These results are in agreement with those of El-Shazly et al. (2020), Da Costa et al. (2017), Talghari et al. (2020) and Bozkurt et al. (2016) who reported that diclazuril, nicarbazin and monensin enhanced productivity of broilers challenged with *Eimeria* species.

The decrease in erythrogram parameters including erythrocytes count and Hb and PCV values in PC birds due to external hemorrhage (blood loss into the gut) resulting from damaged mucosa caused by *Eimeria* multiplication. Dietary administration of anticoccidial compounds particularly in MMD and NN groups improved values of these indices near to that of NC birds (Table 4). The interference of these substances with *Eimeria* replication diminished the external hemorrhage which explains the improvement of these parameters (El-Shazly et al., 2020). Leukogram parameters were markedly affected by *Eimeria* challenge particularly total leukocytes, lymphocytes and heterophils count which were elevated in PC group (Table 5). These results are in line with (Adamu et al., 2013; El-Shazly et al., 2020). According to Irizaary-Rovira (2004), the elevated leukocytes count and differentiation may be associated with intracellular parasitic infections that activate the immune system, particularly the cell-mediated immunity (Al-Taee and Al-Zubaidi, 2017). Lymphocytes have an important role in cellular immunity against *Eimeria* since they accumulated to attack *Eimeria*-infected cells (El-Shazly et al., 2020) and this elucidates the increase in their count in PC birds. The aforementioned parameters were improved by the treatment with anticoccidials and become closer to healthy birds of NC group. This indicates that these substances might indirectly mitigate inflammatory processes related to coccidiosis by hindering the replication of *Eimeria*.

Blood chemistry was greatly affected by *Eimeria* challenge in this study. The decrease in serum TP and globulin and increased AST, ALT, ALP, uric acid, and creatinine levels in PC birds (Table 6) might be due to the malabsorption of protein and other nutrients from the intestine because of hemorrhage and mucosal damage in these birds (Williams, 2005). The reduction in feed intake and absorption of proteins elevates protein catabolism in muscular tissues, muscle degradation and eventually increase serum values of AST, ALT, uric acid and creatinine (El-Shazly et al., 2020). The external hemorrhage may also induce hyperactivity of bone marrow to produce excessive blood corpuscles which elevates serum ALP value (Adamu et al., 2013). In the current study, infestation with coccidian parasites exhibit significant reduction in serum cholesterol, triglycerides, HDL and VLDL levels compared to NC group. Anorexia and clinical anemia may be the reasons for lipid metabolism disorder which decreases values of serum lipid constituents (Freitas et al., 2008; Gautier et al., 2020). Indirect improvement in the aforementioned parameters achieved by the anticoccidial drugs might be as a consequence of the inhibition of *Eimeria* multiplication.

Parasitic infections, such as *Eimeriosis*, has a noxious impact on the microarchitecture of the intestine, leading to ineffective digestion, decrease absorptive surface area and inefficient absorption of nutrients (Adams et al., 1996; Morris et al., 2004; Su et al., 2015; Gottardo et al., 2016). However, these adverse effects on intestinal integrity and gut health may be alleviated using anticoccidial methods of coccidiosis prophylaxis (Bozkurt et al., 2016). Scarce information is available in the literature on the protective effects of single or combined administration of monensin, maduramicin, diclazuril, narasin and nicarbazin on villous structure against *Eimeria spp.* infection in birds. Treated groups of birds showed longer villi and shallower crypts compared to PC birds (Figure 1) which contribute to elevated mucosal digestive enzymes and provide greater surface area for digestion and absorption resulting in effective nutrient transport system (Amat et al., 1996). In our study, dietary supplementation of anticoccidials, particularly in MMD and NN groups, enables broilers to increase villi height and thus compensate for the depression in feed efficiency induced by the infection with *Eimeria spp.* These results are in agreement with those of Bozkurt et al. (2016) and Nabian et al. (2018) who noticed noteworthy recovery in impaired villous structure induced by avian coccidiosis when birds treated with monensin sodium and diclazuril.

The impaired antioxidant status and the occurrence of oxidative stress following *Eimeria spp.* invasion has been reported (Allen, 1997; Georgieva et al., 2006). Antioxidant status on infested birds in the current study was confirmed by both serum content of MDA, catalase, GPx and SOD, and m-RNA expression of GPx and SOD. Contents of MDA and catalase were elevated while SOD and GPx activities were decreased PC birds compared to unchallenged birds (Figure 2). Gene expression of SOD and GPx was increased in PC birds (Figure 3). The elevated serum concentration of MDA in *Eimeria* infected birds could be attributed to the excessive production of ROS caused after *Eimeriosis*, resulting in lipid peroxidation (Allen, 1997; Georgieva et al., 2006; Bun et al., 2011). The enzymatic antioxidant system of chickens with SOD, in most cases of parasitic infection, was remarkably decreased when infected with *Eimeria spp.* (Georgieva et al., 2006). Superoxide dismutase is participating in the systemic antioxidant defense to eliminate and neutralize excessive ROS; thus the reduction in its level is probably due to the elevation in ROS production. The elevation in m-RNA expression of SOD and GPx in PC birds could be attributed to the need for large quantities of these substances to counteract the abundant production of ROS and nitrogen species. Georgieva et al. (2011) reported that the compensatory elevation of catalase following the oxidative stress caused by *Eimeria* challenge was due to impaired ecological oxidative balance and to get rid of excess peroxides. The role of anticoccidials in NS, MMD, and NN groups to restore antioxidative biomarkers near to the normal range of NC could be attributed to their ability to reduce *Eimeria* count in the intestine which improves the antioxidant status of challenged birds.

Antibodies have a crucial role in protective immunity against *Eimeria* protozoa infection and can efficiently hinder growth, development and replication of this parasite in the intestine and thus induce partial protective passive immunity (Anwar et al., 2008; Wallach, 2010). Moreover, Smith et al. (1994) documented positive correlation between protection against coccidiosis and antibody titer. Infection with *Eimeria spp.* also upregulates the expression of proinflammatory cytokines (Lee et al., 2011). These combined cellular and humoral immune responses likely reflect host reactions to infectious *Eimeria* and other pathogens in the gut. In the present study, serum IgA level was elevated and jejunal gene transcripts for IL-6 and IFN-γ were increased in PC birds compared to NC ones (Figures 2 and 3). The therapeutic efficacy of antibiotic ionophores such as monensin, narasin and maduramicin and the synthetic chemicals (nicarbazin and diclazuril) could be due to their impacts on stimulating antibodies production against *Eimeria spp.* Furthermore, these substances are directly cytotoxic for *Eimeria* and would, therefore, be expected to decrease intestinal loads of parasites and pathogenic bacteria resulting in reducing the corresponding host inflammatory responses.

Under the conditions of the present study, all examined anticoccidial drugs were effective in treating Eimeriosis and restoring the measured parameters near to the normal level. The new anticoccidial combination between maduramicin ammonium (Madramycin) and diclazuril (Atozuril) exerted herein pronounced anticoccidial efficacy more than the single administration of monensin sodium (Atomonsin) and can be similar to that of Maxiban72 (narasin and nicarbazin). The current findings encourage future studies to investigate new combinations of existing anticoccidials to overcome the emergence of coccidiosis resistance.
